# Emergence of a novel mobile colistin resistance gene, *mcr-8*, in NDM-producing *Klebsiella pneumoniae*

**DOI:** 10.1038/s41426-018-0124-z

**Published:** 2018-07-04

**Authors:** Xiaoming Wang, Yao Wang, Ying Zhou, Jiyun Li, Wenjuan Yin, Shaolin Wang, Suxia Zhang, Jianzhong Shen, Zhangqi Shen, Yang Wang

**Affiliations:** 1 Beijing Advanced Innovation Center for Food Nutrition and Human Health, College of Veterinary Medicine, China Agricultural University, Beijing, China; 2 Beijing Key Laboratory of Detection Technology for Animal-Derived Food Safety and Beijing Laboratory for Food Quality and Safety, Beijing, China

## Abstract

The rapid increase in carbapenem resistance among gram-negative bacteria has renewed focus on the importance of polymyxin antibiotics (colistin or polymyxin E). However, the recent emergence of plasmid-mediated colistin resistance determinants (*mcr-1*, *-2*, *-3*, *-4*, *-5*, *-6,* and *-7*), especially *mcr-1*, in carbapenem-resistant *Enterobacteriaceae* is a serious threat to global health. Here, we characterized a novel mobile colistin resistance gene, *mcr-8*, located on a transferrable 95,983-bp IncFII-type plasmid in *Klebsiella pneumoniae*. The deduced amino-acid sequence of MCR-8 showed 31.08%, 30.26%, 39.96%, 37.85%, 33.51%, 30.43%, and 37.46% identity to MCR-1, MCR-2, MCR-3, MCR-4, MCR-5, MCR-6, and MCR-7, respectively. Functional cloning indicated that the acquisition of the single *mcr-8* gene significantly increased resistance to colistin in both *Escherichia coli* and *K*. *pneumoniae*. Notably, the coexistence of *mcr-8* and the carbapenemase-encoding gene *bla*_NDM_ was confirmed in *K*. *pneumoniae* isolates of livestock origin. Moreover, BLASTn analysis of *mcr-8* revealed that this gene was present in a colistin- and carbapenem-resistant *K*. *pneumoniae* strain isolated from the sputum of a patient with pneumonia syndrome in the respiratory intensive care unit of a Chinese hospital in 2016. These findings indicated that *mcr-8* has existed for some time and has disseminated among *K*. *pneumoniae* of both animal and human origin, further increasing the public health burden of antimicrobial resistance.

## Introduction

*Klebsiella pneumoniae*, belonging to the family *Enterobacteriaceae*, is a bacterial pathogen commonly associated with nosocomial infections, including pneumonia, bloodstream infection, urinary tract infection, and hepatic abscess, especially among immunocompromised patients^1^. Carbapenem was used as the drug of choice for the treatment of infections caused by multidrug-resistant *K*. *pneumoniae*. However, the increased prevalence of carbapenem-resistant *K*. *pneumoniae* (CRKP) greatly compromised the efficacy of carbapenem antibiotics^2^. Colistin has been considered a last resort antibiotic, used either alone or in combination with other drugs, for the treatment of serious infections caused by CRKP^3^. In recent years, multiple studies have indicated that the prevalence of colistin resistance has increased rapidly among *Enterobacteriaceae*^4^. In 2015, Liu et al.^5^ identified the first mobile colistin resistance gene, *mcr-1*, in *Enterobacteriaceae*, mainly *Escherichia coli* and *K*. *pneumoniae*. In a short time, *mcr-1* and its slightly altered gene variants (*mcr-1.1* to *mcr-1.12*) were ubiquitously identified in various *Enterobacteriaceae* of different origins in over 40 countries across five different continents^6–10^. Several other MCR homologs (MCR-2, MCR-3, MCR-4, and MCR-5) were subsequently identified in *Enterobacteriaceae*. Very recently, two MCR homologs (MCR-6 and MCR-7) were annotated and deposited into GenBank, and the *mcr-7.1* gene was detected in *K*. *pneumoniae* of chicken origin in China^11^. To date, *mcr-2* has only been detected in *E*. *coli* and *Salmonella* isolates from European countries^12,13^, whereas *mcr-3* has been widely identified in *Enterobacteriaceae* (mainly *E*. *coli*) and *Aeromonas* spp. from Asia, Europe, and North America^14^. *mcr-4* and *mcr-5* were first characterized in *Salmonella* and *E*. *coli* from European countries^15,16^ and further detected in *Enterobacter cloacae* and *E*. *coli* isolates from Asia (Singapore and Japan)^17,18^. Thus far, only *mcr-1* and *mcr-3* have been reported in *K*. *pneumoniae*, and the prevalence of these two genes is relatively low^5,6^. In this study, we characterized a novel mobile colistin resistance gene, *mcr-8*, in a *K*. *pneumoniae* isolate and then identified the coexistence of *mcr-8* and *bla*_NDM_ in *K*. *pneumoniae* isolates from both animals and humans.

## Results

### A novel transferrable colistin resistance determinant in *Klebsiella pneumoniae*

During the period from 2015 to 2017, our routine surveillance indicated an increased number of colistin-resistant *K*. *pneumoniae* isolates in which the recently characterized plasmid-mediated colistin resistance genes *mcr-1*, *mcr-2*, *mcr-3*, *mcr-4* and *mcr-5* were absent. One *K*. *pneumoniae* isolate, designated KP91, was obtained from a swine fecal sample. The isolate exhibited a multidrug resistance profile, including resistance to colistin (MIC = 16 μg/ml) (Table 1). Whole-genome sequencing indicated that KP91 belonged to the ST42 group and contained multiple antimicrobial resistance genes, including the β-lactam resistance genes *bla*_SHV-1_ and *bla*_CTX-M-14_, aminoglycoside resistance genes *strA*, *strB*, *armA*, and *aph(4)-Ia*, macrolide resistance genes *mph*(E) and *msr*(E), quinolone resistance genes *oqxA* and *qnrB4*, sulfonamide resistance genes *sul1*, *sul2*, and *sul3*, tetracycline resistance genes *tet*(A), *tet*(B), and *tet*(34), and trimethoprim resistance gene *dfrA12* (Table 1). KP91 also carried multiple virulence genes, including *kfuC*, *mrkABCDF*, *irp2*, *iucA* and *iucB*, which have been associated with urinary tract infections, septicemia, and pneumonia^19–21^.

**Table 1:** Minimum inhibitory concentrations of tested antimicrobial agents for the studied bacterial isolates

Species	MIC (µg/ml)											
	CST	PB	TET	MEM	TAZ	AMC	SXT	AN	GEN	STR	FFC	CIP
*K*.*pneumoniae* KP91	**16**	**8**	**>256**	0.06	**16**	**32/16**	**16**	**>256**	**512**	**256**	**256**	**256**
*E*. *coli* J53 + pKP91	**4**	**4**	1	0.015	0.5	4/2	0.5	1	<0.125	4	4	0.015
*E*. *coli* J53	0.25	0.25	1	0.03	0.5	4/2	0.5	1	<0.125	4	4	0.015
DH5ɑ + pUC19-*mcr-8*	1	0.5	–	–	–	–	–	–	–	–	–	–
DH5ɑ + pUC19	0.25	0.25	–	–	–	–	–	–	–	–	–	–
*K*. *pneumoniae_*	**8**	**8**	–	–	–	–	–	–	–	–	–	–
ATCC13883+pUC19-*mcr-8*												
*K*. *pneumoniae_*	1	2	–	–	–	–	–	–	–	–	–	–
ATCC13883+pUC19												

Antimicrobial susceptibilities, reported as MIC (µg/ml), were interpreted as resistant (bold) or susceptible (plain text) in accordance with established breakpoints. *CST* colistin, *PB* polymyxin B, *TET* tetracycline, *MEM* meropenem, *TAZ* ceftazidime, *AMC* amoxicillin-clavulanate, *SXT* trimethoprim-sulfamethoxazole, *AN* amikacin, *GEN* gentamycin, *STR* streptomycin, *FFC* florfenicol, *CIP* ciprofloxacin. “–” indicates that the MIC was not measured

**Fig. 1:**
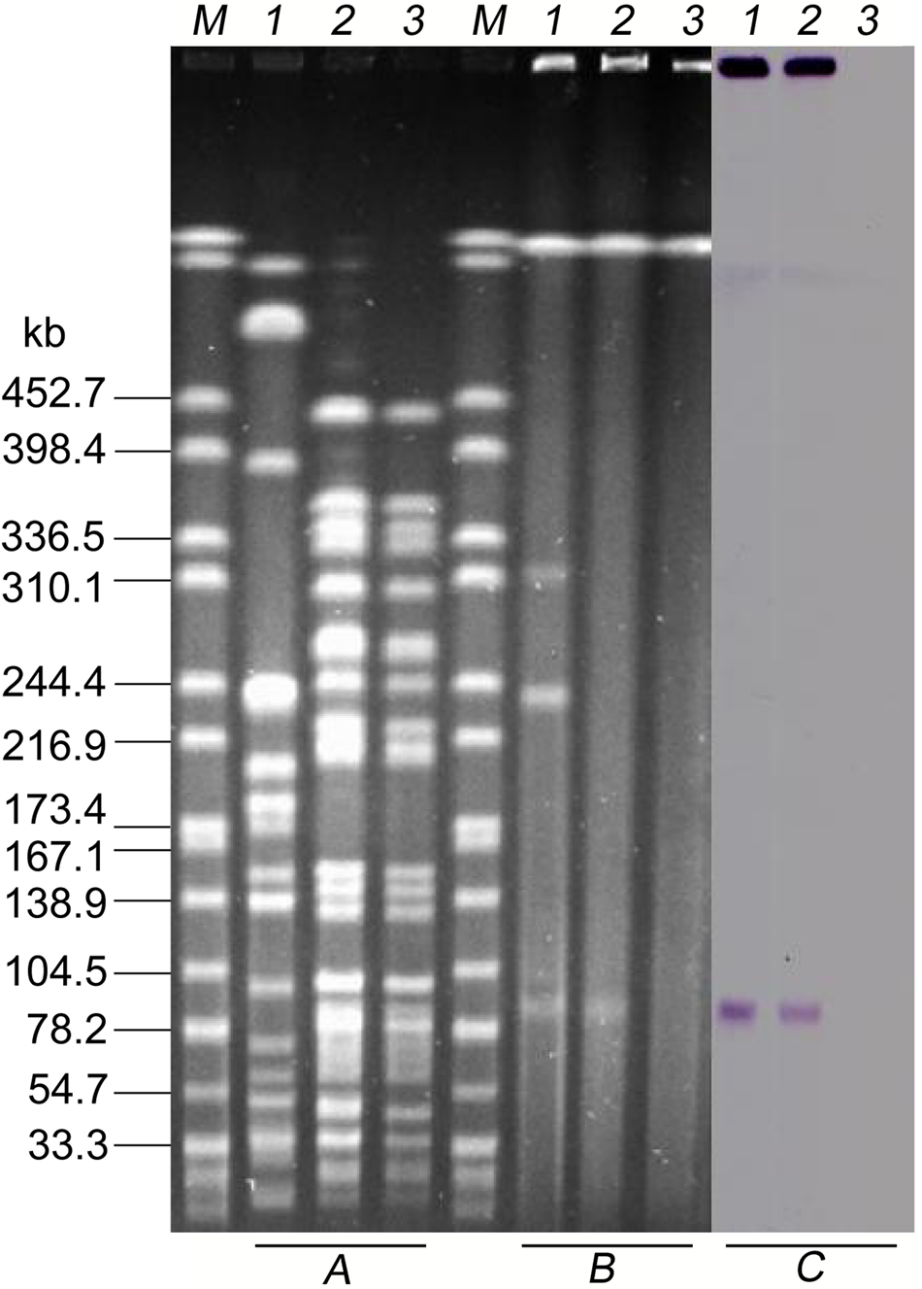
Location of *mcr-8* in *Klebsiella pneumoniae* KP91 and its transconjugants. **a** *Xba*I-digested PFGE of *K*. *pneumoniae* KP91, transconjugants, and the recipient *E*. *coli* strain J53. **b** S1-PFGE and **c** the corresponding Southern hybridization using the *mcr-8*-specific probe. Lane M, marker H9812; lane 1, *K*. *pneumoniae* KP91; lane 2, transconjugant *E*. *coli* J53-pKP91; lane 3, recipient *E*. *coli* J53

Conjugation assays showed that the colistin resistance determinant in *K*. *pneumoniae* KP91 can be transferred into the recipient *E*. *coli* strain J53. The transconjugant J53-pKP91 had a pulsed-field gel electrophoresis (PFGE) pattern identical to that of *E*. *coli* J53 but distinct from that of KP91. S1-PFGE indicated that J53-pKP91 differed from *E*. *coli* J53 by the presence of a single ~90-kb plasmid, designated pKP91 (Fig. 1). These results confirmed the presence of an uncharacterized colistin resistance determinant located on a conjugative plasmid in *K*. *pneumoniae* KP91.

### Identification and functional confirmation of *mcr-8* in *K*. *pneumoniae* and *E*. *coli*

**Fig. 2:**
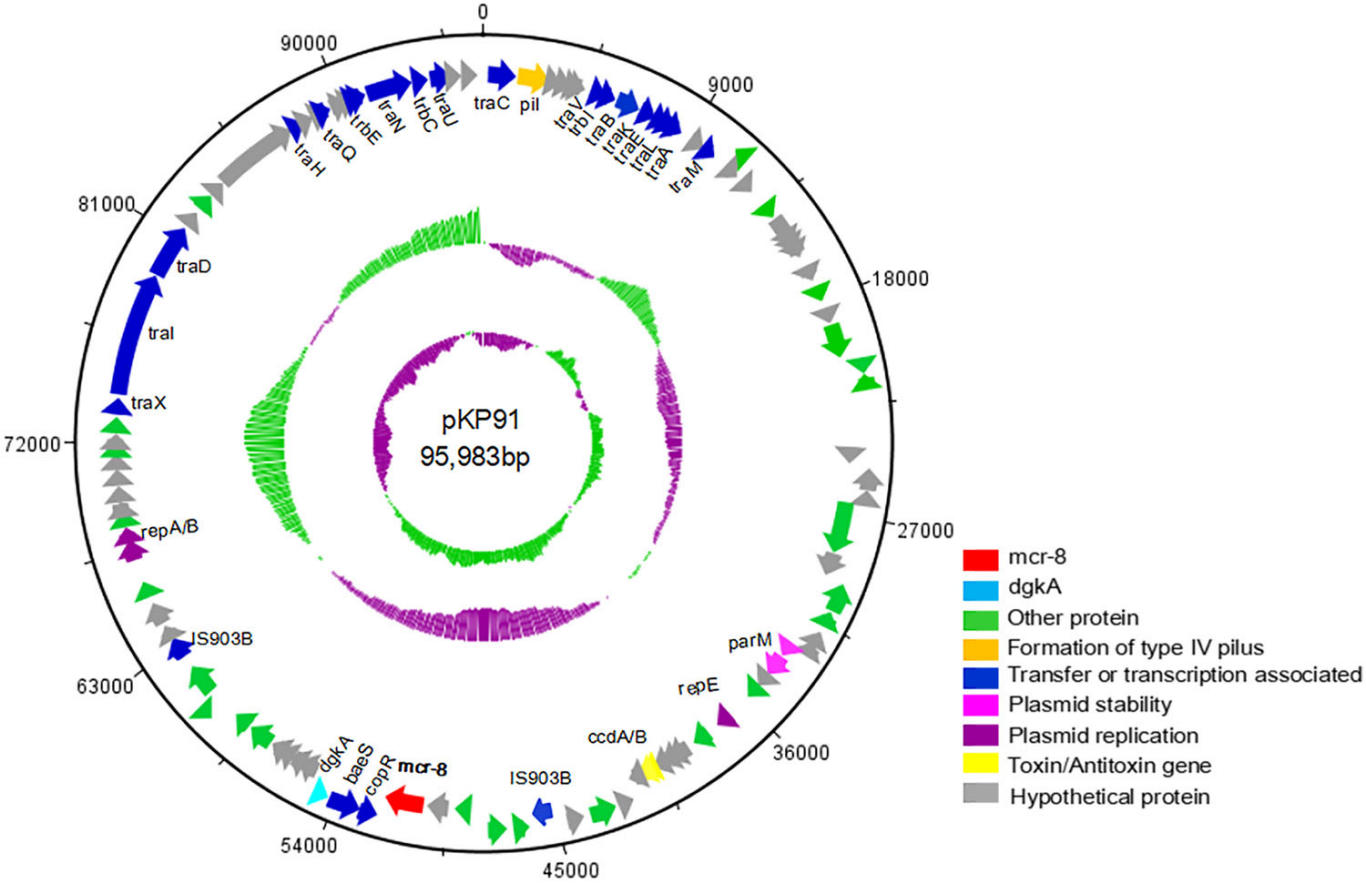
The genetic contents of plasmid pKP91. Circles display (outside to inside) (i) size in bp and (ii) the positions of predicted coding sequences transcribed in a clockwise orientation

**Fig. 3:**
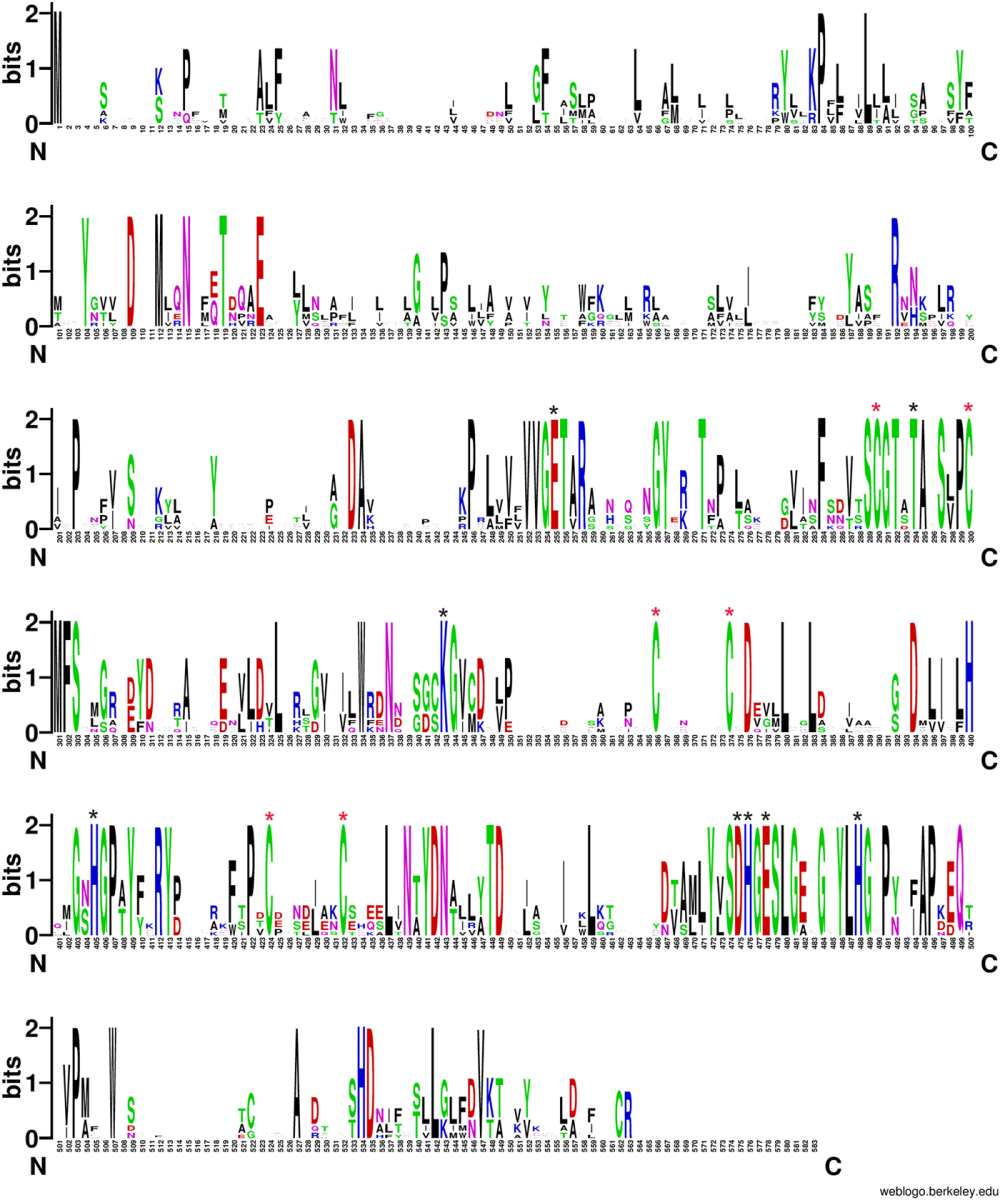
WebLogo of amino-acid sequences of MCR-1–8. A logo represents each column of the alignment in a stack of letters, with the height of each letter proportional to the observed frequency of the corresponding amino acid, and the overall height of each stack proportional to the sequence conservation, measured in bits, at that position. Red asterisks indicate the six conserved cysteine residues that form three disulfide bonds. Black asterisks indicate the conserved active sites among MCR-1–8. The logo was built using WebLogo software (http://weblogo.berkeley.edu/logo.cgi)

To characterize the colistin resistance determinants, we used Single Molecule Real-Time (SMRT) sequencing to generate the complete sequence of the plasmid isolated from transconjugant J53-pKP91. SMRT sequencing revealed that plasmid pKP91 was 95,983 bp in size, with a GC content of 50.16%, which differed from the GC content of the genome (56.33%) (Fig. 2). pKP91 was identified as a typical IncFII-type plasmid and contained genes involved in plasmid stability, formation of a type-IV pilus, and conjugative transfer (Fig. 2). BLASTn analysis identified a 1698-bp open-reading frame (ORF) encoding a putative phosphoethanolamine transferase, which showed 50.23% nucleotide sequence identity to the corresponding region of *mcr-3*. The amino-acid sequence of this ORF showed 31.08%, 30.26%, 39.96%, 37.85%, 33.51%, 30.43%, and 37.46% amino-acid sequence identity to MCR-1, MCR-2, MCR-3, MCR-4, MCR-5, MCR-6 and MCR-7, respectively (Fig. S1). Sequence alignment indicated the previously identified active sites (E246, T285, K333, H395, D465, H466, E468, H478) and six cysteine residues that form three disulfide bonds between residues Cys281/Cys291, Cys356/Cys364, and Cys414/Cys422 in the catalytic domain of MCR-1 are highly conserved among these protein ORFs (Fig. 3, Fig. S1)^22–25^. These results suggested that the corresponding gene might encode a functional phosphoethanolamine transferase that mediates resistance to colistin in both *K*. *pneumoniae* and its *E*. *coli* transconjugant. In addition, the expression of this single ORF in *E*. *coli* DH5ɑ and *K*. *pneumoniae* ATCC13883 resulted in four- and eightfold increases, respectively, in the minimum inhibitory concentration of colistin (Table 1), which further confirmed the function of the gene. This gene was designated *mcr-8* based on the paper regarding the naming or assignment of allele numbers for mobile colistin resistance (*mcr*) genes (Partridge, S. et al Proposal for assignment of allele numbers for mobile colistin resistance (*mcr*) genes. *J*. *Antimicrob*. *Chemother*) (under revision).

### The presence of *mcr*-*8* in *K*. *pneumoniae* of human origin

BLASTp analysis of MCR-8 against the NCBI database revealed a protein sequence with 99% amino-acid identity and 99% query coverage to MCR-8 in a clinical CRKP strain WCHKP1845, originally isolated from the sputum of a patient with pneumonia in the respiratory intensive care unit at West China Hospital, Chengdu, China, in May 2016. In the corresponding paper, the authors claimed that colistin resistance was conferred by an unknown mechanism in strain WCHKP1845^26^. These analyses suggest that *mcr-8* may be responsible for the colistin resistance of this strain, and the clinical isolate WCHKP1845 with ST1 was not related to the pig-origin KP91 with ST42.

### Structure prediction and genetic environment analysis of MCR-8

**Fig. 4:**
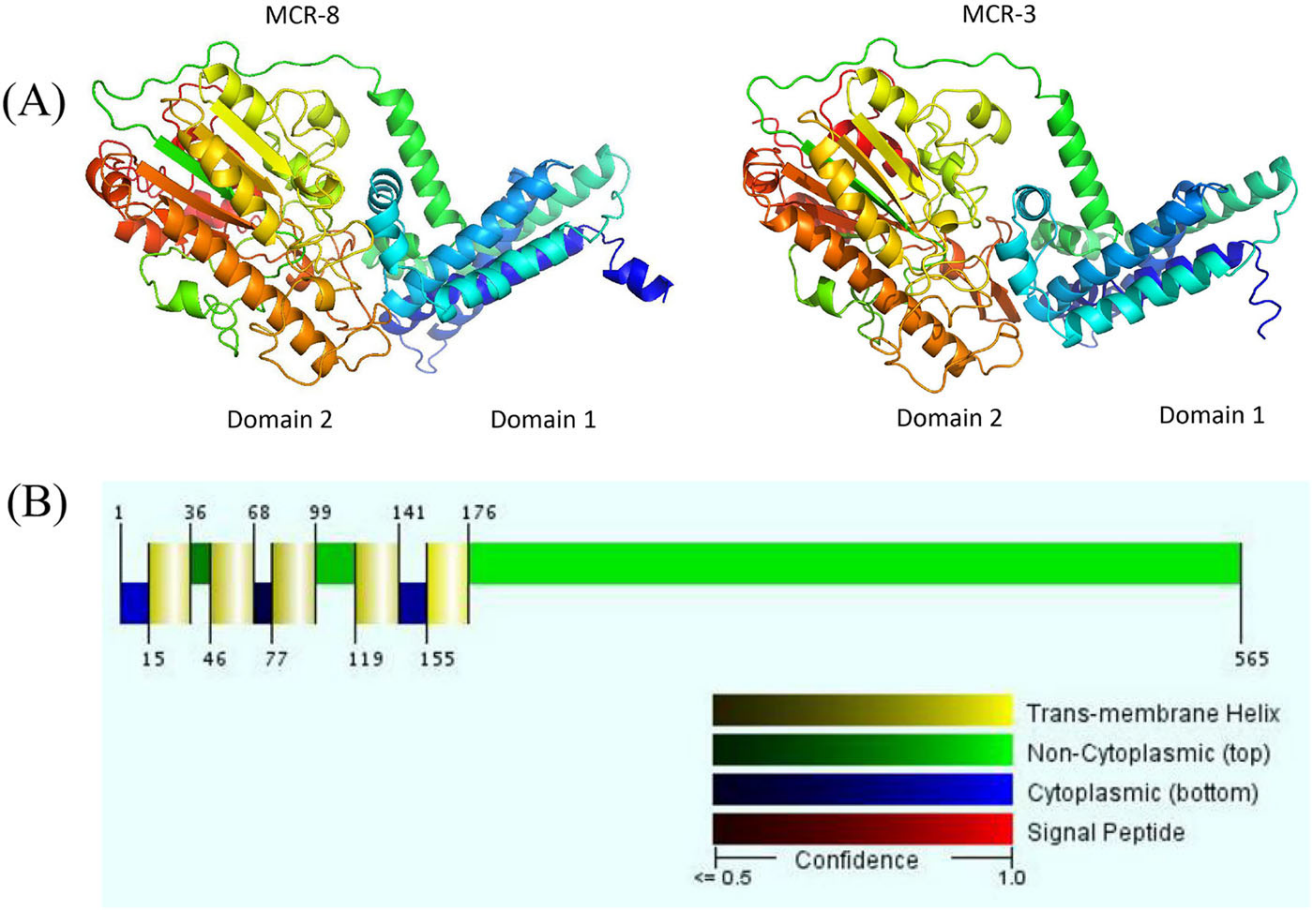
Predicted crystal structure and transmembrane domain of MCR-8. **a** Structure prediction for MCR-8 and reference protein MCR-3. Domain 1 was predicted to be a transmembrane domain, while domain 2 was predicted to be a phosphoethanolamine transferase. **b** The five transmembrane α-helices predicted by the Philius transmembrane prediction server (type confidence, 0.99; topology confidence, 0.88)

Similar to previously characterized MCR proteins, MCR-8 was predicted using RaptorX (Xu Group, Chicago, IL, USA) to contain two domains (Fig. 4a). Domain 1 (residues 1–234) contained five transmembrane α-helices (Fig. 4b), whereas domain 2 (residues 235–565) contained the putative catalytic center. i-Tasser homology modeling revealed that the best-fit structure in the Protein Data Bank for MCR-8 was 5FGN, which was the first characterized full-length lipid A phosphoethanolamine transferase from *Neisseria meningitidis*^27^.

**Fig. 5:**
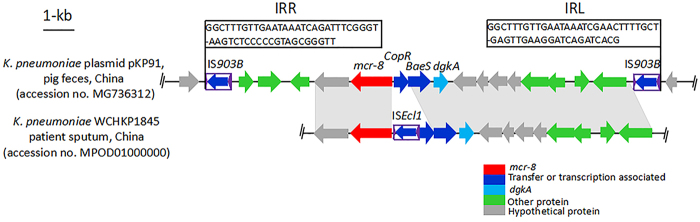
Comparison of the genetic environments of *mcr-8*. *K*. *pneumoniae* WCHKP1845 was isolated from the sputum of patients in China (accession no. MPOD01000000). The positions and orientations of the genes are indicated by arrows, with the direction of transcription shown by the arrowhead. Gray shading indicates > 90% nucleotide sequence identity

Genetic environment analysis revealed that two complete insertion sequences of IS*903B*, originating from *E*. *coli*, were located up- and downstream of *mcr-8* in pKP91. In addition, the 50-bp IRR and 50-bp IRL (Fig. 5) were identical to those surrounding IS*903B* described in the ISfinder database (https://www-is.biotoul.fr/index.php). IS*903B* was absent from the 13.47-kb NCBI contig from clinical WCHKP1845, whereas a complete insertion sequence, IS*Ecl1*, originating from *Enterobacter cloacae*, was present downstream of *mcr-8* in WCHKP1845 (Fig. 5). These findings revealed that several IS elements (IS*903B*, IS*Ecl1*) may play a pivotal role in the dissemination of *mcr-8* among *Enterobacteriaceae*.

### Coexistence of *mcr-8* and carbapenemase genes in *K*. *pneumoniae* isolates

In total, we examined 53 colistin- and carbapenem-resistant *K*. *pneumoniae* isolates derived from pigs (*n* = 28) and chickens (*n* = 25). Of these, four (pig = 3, chicken = 1) isolates were positive for *mcr-8*, whereas five (pig = 3, chicken = 2) were positive for *mcr-1*. In addition, we detected the presence of *bla*_NDM_ but not the *bla*_VIM_, *bla*_IMP_, *bla*_OXA-48_, or *bla*_KPC_ genes in 47 of the carbapenem-resistant *K*. *pneumoniae* isolates. *mcr-8* and *bla*_NDM_ were shown to coexist in three isolates. These findings indicated that the novel *mcr-8* gene has disseminated among *K*. *pneumoniae* and that the prevalence and coexistence of *mcr-8* and *bla*_NDM_ in *K*. *pneumoniae* may further threaten public health through either the food chain or environmental routes.

### Phylogenetic typing analysis

**Fig. 6:**
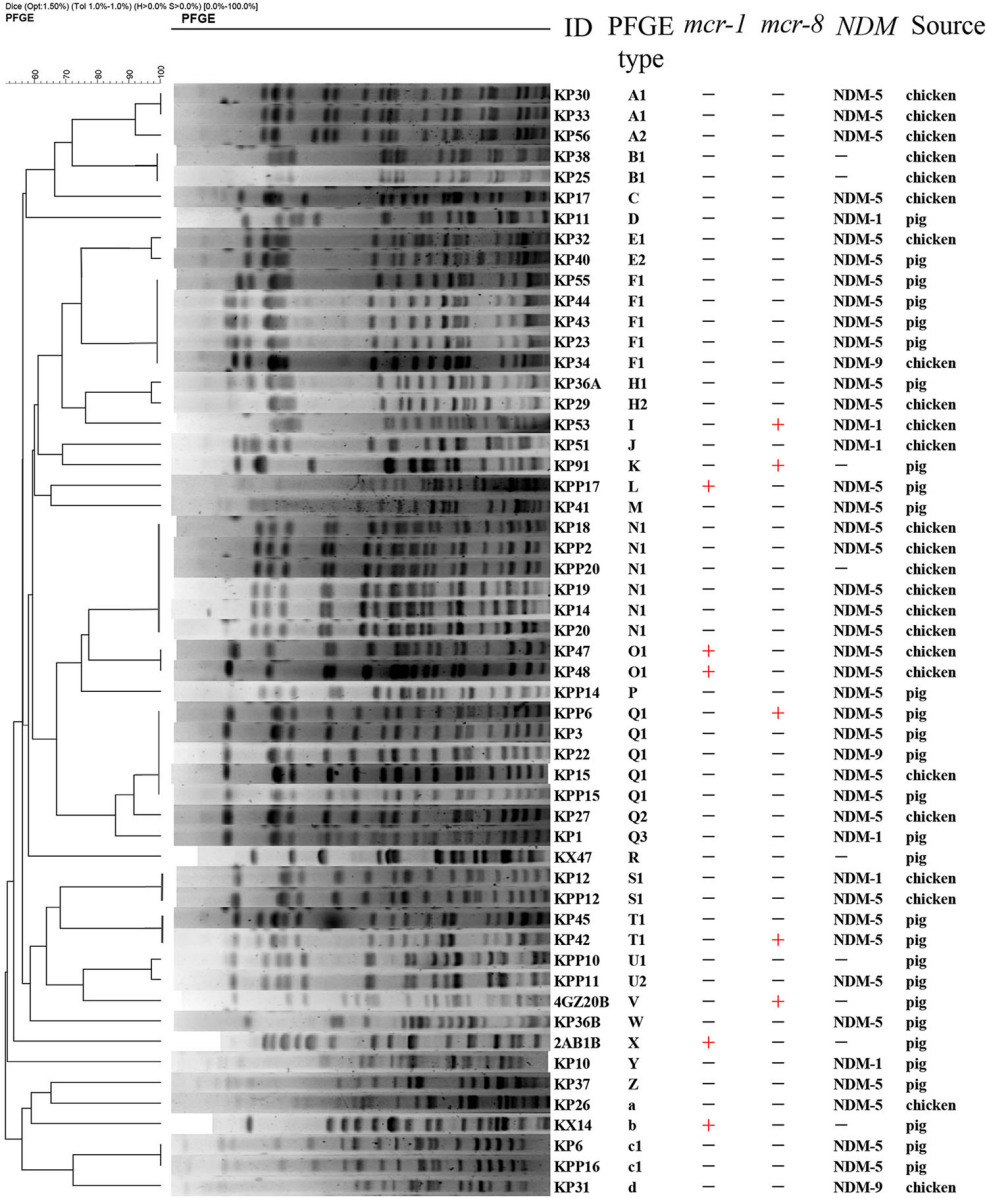
PFGE analysis of *mcr-1-* and *mcr-8*-positive *Klebsiella pneumoniae* isolates as well as other *K*. *pneumoniae* isolates. *Xba*I was used for digestion of the genomic DNA. All *K*. *pneumoniae* isolates were collected from pig fecal matter and chicken cloacae samples from Shandong, China

To determine whether the *mcr-1-* and *mcr-8*-positive *K*. *pneumoniae* isolates were genetically related, five *mcr-1-* and five *mcr-8*-positive isolates, including KP91, along with 44 *mcr*-negative carbapenem-resistant *K*. *pneumoniae* isolates, were selected for PFGE analysis with *Xba*I digestion. Using a cutoff of 80% pattern similarity, the 54*K*. *pneumoniae* isolates were grouped into 13 clusters (PFGE patterns represented by multiple strains) and 17 unique PFGE patterns (PFGE patterns represented by a single strain) (Fig. 6). Overall, the *K*. *pneumoniae* isolates from different regions were genetically diverse, suggesting that the *mcr-8*-positive *K*. *pneumoniae* isolates were also genetically diverse and that *mcr-8* could disseminate among different *K*. *pneumoniae* isolates, mainly by horizontal transmission.

## Discussion

The rapid emergence of mobile colistin resistance genes represents a paradigm shift in colistin-resistant mechanisms, which were long dominated by chromosomal mutations and vertical transmission. To date, *mcr-1* and *mcr-3* have frequently been detected in *Enterobacteriaceae* from different regions^28^, whereas *mcr-2*, *mcr-4*, and *mcr-5* have been rarely reported. Although all of these *mcr* genes have been identified in various *Enterobacteriaceae* and non-*Enterobacteriaceae* species, *E*. *coli* remains the predominant host. A small number of reports have indicated the presence of *mcr-1* and *mcr-3* in *K*. *pneumoniae* at relatively low detection rates^6,18,29^. In this study, we identified a novel mobile colistin gene, *mcr-8*, carried on an IncFII-type conjugative plasmid in *K*. *pneumoniae*. The presence of *mcr-8* (7.54%, 4/53) among the colistin-resistant CRKP isolates indicates that this novel mobile colistin resistance gene may already be widely disseminated among *K*. *pneumoniae* isolates of livestock origin.

**Fig. 7:**
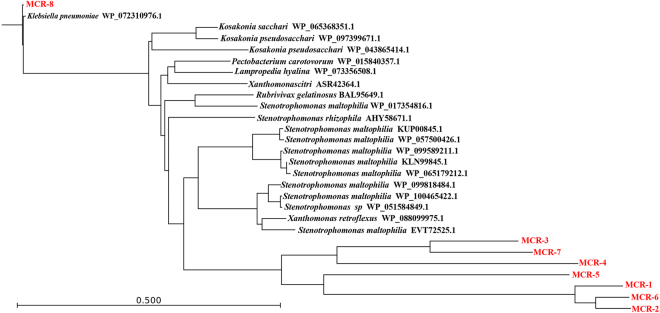
Phylogenetic tree of the deduced amino-acid sequences of 20 putative phosphoethanolamine transferases from different bacterial species and MCR-1–7 with MCR-8 using CLC Genomics Workbench 9 (CLC Bio-Qiagen, Aarhus, Denmark)

MCR-8 homologs are present in various other species. BLASTp analysis also indicated that a number of proteins in the NCBI database showed 60–70% amino-acid identity to MCR-8. These putative phosphoethanolamine-lipid A transferase proteins include sequences from *Kosakonia sacchari* (WP_065368351.1), *Kosakonia pseudosacchari* (WP_097399671.1), *Klebsiella aerogenes* (WP_043865414.1), *Pectobacterium carotovorum* (WP_015840357.1), *Lampropedia hyalina* (WP_073356508.1) *Acidovorax avenae* subsp. *avenae* (WP_013595040.1), *Rubrivivax gelatinosus* (WP_088099975.1), two *Xanthomonas* isolates, and 11 *Stenotrophomonas* sp. isolates (Fig. 7, Table S2). The majority of these bacteria are pathogens of plants, including sweet potato, sugarcane root, pumpkin, carrot, rice, potato, rapeseed, and shrub willow, but some of the strains were isolated from manure, soil, and cerebrospinal fluid. In total, the bacteria came from 15 countries across Asia, Europe, South America, and Oceania. These findings indicate that MCR-8-like phosphoethanolamine transferases are widely distributed among various bacteria from different environments and pose a potential risk of transfer to human pathogens.

In conclusion, we identified a novel gene, *mcr-8*, conferring resistance to colistin, which is considered the last resort drug for the treatment of carbapenem-resistant *Enterobacteriaceae*, especially CRKP. The coexistence of *mcr-8* and *bla*_NDM_ was noted in *K*. *pneumoniae* from both food-producing animals and human clinical samples and poses a serious public health concern. Therefore, further surveillance may help us understand the prevalence and dissemination of this novel antibiotic resistance gene.

## Materials and methods

### Bacterial isolation and susceptibility testing

All *K*. *pneumoniae* isolates were recovered from pig fecal matter and chicken cloacae samples collected from Shandong Province, China, according to a previously described protocol^30^. Isolates were identified as described previously^27^. The MICs of the *K*. *pneumoniae* isolates to colistin, tetracycline, meropenem, ceftazidime, amoxicillin-clavulanate, trimethoprim-sulfamethoxazole, amikacin, gentamicin, streptomycin, florfenicol, and ciprofloxacin were determined using the broth microdilution method according to the guidelines of the Clinical and Laboratory Standards Institute (document VET01-A4), and the European Committee on Antimicrobial Susceptibility Testing (EUCAST) (http://www.eucast.org).

### Conjugation assay

Conjugation assays were performed according to a previously described method^31^. In brief, the azide-resistant *E*. *coli* strain J53 was used as the recipient, and donor and recipient strains were mixed at a ratio of 1:3 on a microporous membrane for 12 h. The mixtures were collected and then plated on Luria-Bertani agar containing azide (100 μg/ml) and colistin (2 μg/ml).

### Genome sequencing and assembly

Genomic DNA was extracted from *K*. *pneumoniae* isolate KP91 using a Wizard Genomic DNA Purification Kit (Promega, Beijing, China) and used as template for 150-bp paired-end whole-genome sequencing using the Illumina HiSeq 2500 System (Annoroad, Beijing, China). The draft genome was assembled using the CLC Genomics Workbench 9.0 (CLC Bio-Qiagen, Aarhus, Denmark). The conjugative plasmid was extracted from *E*. *coli* transconjugant strain J53-pKP91 using a Plasmid Midi Kit (OMEGA, Norcross, GA, USA) and then sequenced using both the Illumina HiSeq platform and the PacBio RSII System (Sinobiocore, Beijing, China). Plasmid assembly was performed using the Hierarchical Genome Assembly Process (HGAP) and Quiver as part of the SMRT analysis program (version 2.3) using the HGAP3 protocol and then corrected using Pilon.

### Functional cloning of *mcr-8*

A 2631-bp DNA fragment including the *mcr-8* coding sequence and its flanking regions was amplified from KP91 using the primers CMCR8-F (5′-CCCAAGCTTTTGATTGTCCCTGTCGCCAT-3′) and CMCR8-R (5′-CACCGATAAGAGGAACCAGTGAATTCCGG-3′) and then ligated into cloning vector pUC19 to yield pUC19-*mcr-8*. The recombinant vector was then transferred into *E*. *coli* DH5α and *K*. *pneumoniae* ATCC13883 via electroporation.

### Sequence analysis and structure prediction of MCR-8

Plasmid replicon typing and multilocus sequence typing were carried out using tools available from the Center for Genomic Epidemiology website (http://whileereas genomicepidemiology.org/), whereas coding sequence prediction was completed using Prodigal (http://compbio.ornl.gov/prodigal/). Genomic sequences were annotated using the RFam (http://rfam.xfam.org), nr (http://www.ncbi.nlm.nih.gov/RefSeq/), KEGG (http://www.genome.jp/kegg/), and SwissProt databases^32^. Reference sequences of antibiotic resistance genes were obtained from the ARG-ANNOT database^33^. Prediction of the MCR-8 structure was carried out using i-Tasser homology modeling (https://zhanglab.ccmb.med.umich.edu/I-TASSER/), and the structure was depicted in cartoon form using PyMOL software 1.6.0 (PyMOL, DeLano Scientific LLC, San Carlos, America). Transmembrane α-helices were predicted using the Philius transmembrane prediction server (http://yeastrc.org/philius/showResults.do).

### Detection of genes conferring resistance to colistin and carbapenem

The presence of the five colistin resistance genes, *mcr-1* to -*5*, in the *K*. *pneumoniae* isolates was determined by PCR and Sanger sequencing. We also detected the presence of the carbapenem resistance genes *bla*_NDM_, *bla*_VIM_, *bla*_IMP_, *bla*_OXA-48_, and *bla*_KPC_ in the *K*. *pneumoniae* isolates by PCR analysis. The primers and conditions used for the PCR assays are listed in Table S1.

### PFGE, S1 nuclease-PFGE, and Southern blotting

PFGE analysis of *K*. *pneumoniae* isolates was performed using *Xba*I as the restriction endonuclease, as previously described^34,35^. *Salmonella enterica* serovar Braenderup H9812 digested with *Xba*I was used as the reference marker. The PFGE results were analyzed using InfoQuest software version 4.5 (Bio-Rad Laboratories, Hercules, CA, USA). S1 nuclease-PFGE was performed to determine the plasmid profiles. The genomic location of *mcr-8* was indicated by Southern hybridization using a digoxigenin-labeled *mcr-8* probe according to the manufacturer’s instructions for the DIG-High Prime DNA Labeling and Detection Starter Kit II (Roche Diagnostics, Basel, Switzerland).

### Accession number

The complete nucleotide sequence of plasmid pKP91 has been deposited in GenBank under accession no. MG736312.

## Electronic supplementary material

Figure S1 and Table S1 and S2
