# Supplementary material for: Emergence of a novel mobile colistin resistance gene, mcr-8, in NDM-producing Klebsiella pneumoniae
Source: Emerg Microbes Infect. 2018 Jul 4;7:122. doi: 10.1038/s41426-018-0124-z (PMC6030107; doi:10.1038/s41426-018-0124-z)
Supplement: Supplementary file 1 — Figure S1 and Table S1 and S2 [file 41426_2018_124_MOESM1_ESM.pdf]

Table S1 PCR primers and contidions used in this study.

Primers	Sequence(5'-3')	Annealing Temperature(℃)	Product Size(bp)	Reference
MCR-1F	ATCAGCCAAACCTATCCCATCG	55	1257	This study
MCR-1R	GCAGACGCACAGCAATGCCTAT			
MCR-2F	GCGATGGCGGTCTATCCTGTAT	55	378	This study
MCR-2R	TGCGATGACATGGGGTGTCAGC			
MCR-3F	TATGGGTTACTATTGCTGG	55	814	This study
MCR-3R	CTACCCTGATGCTCATCG			
MCR-4F	GTCATAGTGGTATAAAAGTACAG	55	669	This study
MCR-4R	CCACCGTCTATCAGAGCCAAC			
MCR-5F	GCGGTTGTCTGCATTTATCAC	55	1042	This study
MCR-5R	CTTTGAAAACCTGTCTTCGGCA			
MCR-6F	GTCCGGTCAATCCCTATCTGT	55	556	This study
MCR-6R	ATCACGGGATTGACATAGCTAC			
MCR-7F	TGCTCAAGCCCTTCTTTTCGT	55	892	This study
MCR-7R	TTCATCTGCGCCACCTCGT			
MCR-8F	AACCGCCAGAGCACAGAATT	60	667	This study
MCR-8R	TTCCCCCAGCGATTCTCCAT			
NDM-F	GGTTTGGCGATCTGGTTTTC	55	621	1
NDM-R	CGGAATGGCTCATCACGATC			
KPC-F	CGTCTAGTTCTGCTGTCTTG	55	566	1
KPC-R	CTTGTCATCCTTGTTAGGCG			
VIM-F	GATGGTGTTTGGTCGCATA	55	390	1
VIM-R	CGAATGCGCAGCACCAG			
OXA-F	GCGTGGTTAAGGATGAACAC	55	438	1
OXA-R	CATCAAGTTCAACCCAACCG			
IMP-F	GGAATAGAGTGGCTTAAYTCTC	55	232	1
IMP-R	GGTTTAAYAAAACAACCACC			

1. Poirel, L. et al. Multiplex PCR for detection of acquired carbapenemase genes. *Diagn. Micr. Infec. Dis.* 70, 119-23 (2011).

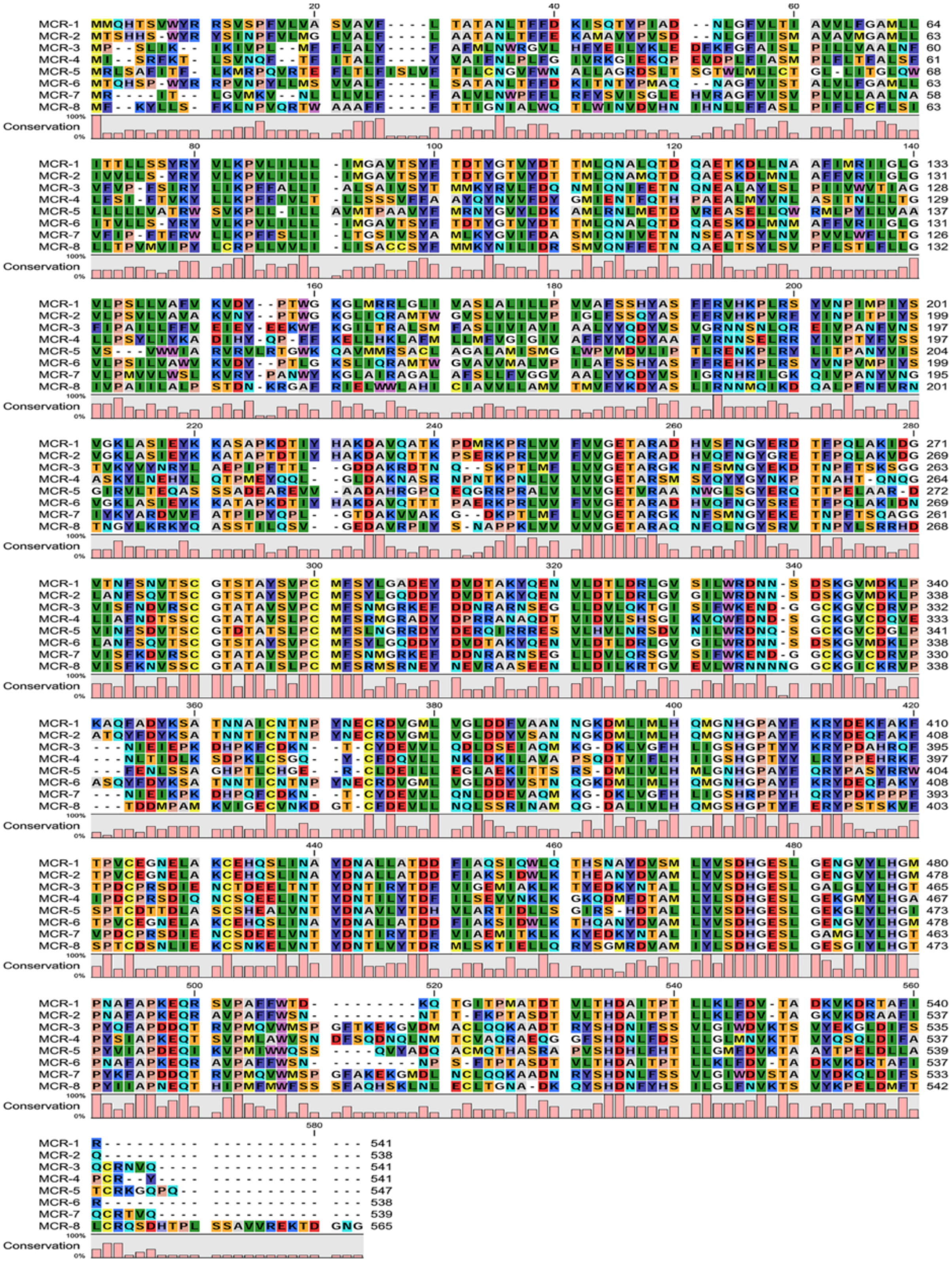

Figure S1. Alignment of the predicted amino acid sequences of MCR-1, MCR-2, MCR-3, MCR-4, MCR-5, MCR-6, MCR-7 and MCR-8, and created using CLC Genomics Workbench 9 (CLC Bio-Qiagen, Aarhus, Denmark).

Table S2. Information of *mcr-6* and *mcr-6*-like genes and their deduced MCR-6 and MCR-6-like proteins collected from GenBank database

Strain	Nucleotide ID	Nucleotide identity with *mcr-6*	Protein ID	Protein identity with MCR-6	Country	Sample source	Date of sample collection/data submission (yyyy/mm/dd)
Klebsiella pneumoniae	NZ_MPOD01000035.1	99.7%	WP_072310976.1	99%	China	Human sputum	2016/5
Kosakonia sacchari	NZ_CP016337.1	64.2%	WP_065368351.1	70%	Japan	Sweet Potato	2017/4/13
Kosakonia pseudosacchari	NZ_NITV01000001.1	63.6%	WP_097399671.1	70%	China	Sugarcane roots	2017/10/13
Klebsiella aerogenes	NZ_JXTQ01000011.1	61.9%	WP_043865414.1	66%	Belgium	Cucurbita pepo	2017/4/06
Pectobacterium carotovorum	NC_012917.1	64.2%	WP_015840357.1	69%	United States	Carrot	2017/3/30
Lampropedia hyalina	NZ_FQUZ01000022.1	63.7%	WP_073356508.1	68%	United States	Unknown	2017/4/17
Xanthomonas citri	CP016836.1	60.7%	ASR42364.1	65%	China	Mango	2017/8/3
Xanthomonas retroflexus	NZ_FRDD01000007.1	56.4%	WP_088099975.1	60%	Denmark	Unknown	2017/6/16
Rubrivivax gelatinosus	AP012320.1	58.9%	BAL95649.1	65%	Japan	Unknown	2016/10/7
Stenotrophomonas maltophilia	NZ_PEJT01000001.1	57.7%	WP_099818484.1	62%	Germany	Rice paddy	2017/11/09
	NZ_LZPC01000026.1	55.2%	WP_065179212.1	62%	France	Manures	2017/4/5
	NZ_MQWX01000061.1	55.1%	WP_099589211.1	61%	Italy	Human lung	2017/11/5
	NZ_NEQS01000012.1	57.3%	WP_100465422.1	61%	Spain	Potato	2017/12/3
	LQNV01000616.1	54.4%	KUP00845.1	61%	Brazil	Human blood	2016/1/15
	AZAE01000001.1	57.0%	EVT72525.1	60%	Czech Republic	Soil	2014/2/04
	LDAV01000276.1	55.4%	KLN99845.1	61%	Saudi Arabia	Water	2015/06/5
	NZ_LLXW01000101.1	55.01%	WP_057500426.1	61%	India	Cerebrospinal fluid	2016/06/3
	NZ_ALYK02000025.1	60.4%	WP_017354816.1	63%	China	Human sputum	2017/4/11
Stenotrophomonas rhizophila	CP007597.1	59.6%	AHY58671.1	64%	Austria	Brassica napus	2014/5/1
Stenotrophomonas sp	NZ_JFYS01000001.1	56.5%	WP_051584849.1	60%	Malaysia	Shrub willow	2017/4/9
